# Reply: The value of case–control audits of screening

**DOI:** 10.1038/sj.bjc.6601599

**Published:** 2004-03-02

**Authors:** P Sasieni, J Adams, J Cuzick

**Affiliations:** 1 Cancer Research UK Department of Epidemiology, Mathematics & Statistics, Wolfson Institute of Preventive Medicine, Queen Mary College, Charterhouse Square, London EC1M 6BQ, UK

**Sir**,

Dr Herbert makes a number of points about our paper that we shall address in turn. Her basic thesis is that when coverage is high, comparison of cervical smear histories in women with and without cancer does not provide useful estimates of the effectiveness (or even the relative effectiveness) of screening at different ages. We strongly disagree. Although observational studies are subject to bias, we do not believe that this can explain the substantial differences in effectiveness that we observed across different age groups. The percentage of women with a screening smear is almost identical in cases and controls aged 20–29 years (74.3 *vs* 74.8%), underlying the lack of effectiveness of screening in this group compared to women aged 45–64 years, in whom coverage is much greater among controls (81.5 *vs* 59.0%).

We agree that some of the screening in our study dates back to the late 1980s and that a variety of quality assurance measures have been introduced since. However, in general, one would expect any advantage of 3-yearly screening over 5-yearly screening to diminish as the quality of screening improves. Table 1b

**Table 1:** Odds ratios for fully invasive cervical cancer by time since last operationally negative cytological smear

	**Odds ratio (95% CI)**		
**Age**	**20–39 (*n*=438)**	**40–54 (*n*=481)**	**55–69 (*n*=386)**
**(a) All data**			
No previous negative	1.0*	1.0*	1.0*

Time since last negative smear			
0–2.9	0.28 (0.20–0.41)	0.12 (0.08–0.17)	0.13 (0.08–0.19)
3.0–4.9	1.03 (0.68–1.56)	0.39 (0.26–0.58)	0.20 (0.12–0.33)
Over 5.0	2.05 (1.20–3.49)	0.72 (0.43–1.18)	0.45 (0.25–0.81)

**Age**	**20–39 (*n*=183)**	**40–54 (*n*=229)**	**55–69 (*n*=160)**
**(b) Women diagnosed since 1995**			
No previous negative	1.0*	1.0*	1.0*

Time since last negative smear			
0–2.9	0.28 (0.16–0.51)	0.08 (0.05–0.15)	0.11 (0.06–0.20)
3.0–4.9	1.34 (0.70–2.57)	0.34 (0.18–0.62)	0.19 (0.09–0.42)
Over 5.0	2.80 (1.26–6.22)	0.65 (0.31–1.36)	0.35 (0.15–0.79)

**Age**	**20–39 (*n*=221)**	**40–54 (*n*=260)**	**55–69 (*n*=225)**
**(c) Data restricted to Health Authorities with staging information for all women**			
No previous negative	1.0*	1.0*	1.0*

Time since last negative smear			
0–2.9	0.32 (0.19–0.53)	0.09 (0.05–0.16)	0.10 (0.06–0.18)
3.0–4.9	0.96 (0.52–1.74)	0.34 (0.19–0.59)	0.18 (0.10–0.34)
Over 5.0	1.81 (0.88–3.75)	0.78 (0.38–1.60)	0.33 (0.16–0.69)

* Reference group.

provides an analysis restricted to cases (and their controls) diagnosed since January 1995. The results are quantitatively similar (and qualitatively unchanged) to those presented in our paper (Table 1a).

Although it is true that screening coverage improved considerably between 1987 and 1993, the claim that, in 1994–1995, over 90% of women had been screened is erroneous. This figure is based on the proportion who had been (or were due to be) sent an invitation, not the proportion actually screened. No data exist for the proportion of women aged 25–64 years in 1994 who had ever been screened. However, it is unlikely to be that much greater than the proportion screened in the previous 5 years (85.7%), since coverage was low prior to 1990 and women screened then were likely to return for screening when invited. Additionally, the relevance of this to comparing 3- *vs* 5-yearly screening is dubious.

We do not believe that cases with unknown stage were more likely to be fully invasive. Most Health Authorities provided information on stage from all or none of the cases for a given year. Restricting analysis to sources that provided complete staging on all cases submitted made little difference to the results (Table 1c).

We agree that due to the poor sensitivity of cervical cytology, the number of previous recent smears is also important. This was illustrated in Tables 3 and 4, in which the added affect of having more than one previous smear was estimated. The results further support our main finding that screening is less effective in younger women. The additional relative benefit of a second negative smear in women aged 20–39 years was only 1.03 compared to 1.32 and 1.35 for women aged 40–54 and 55–69 years, respectively.

Dr Herbert points out that in 1998 over 4000 cases of CIN3 were detected in UK women under 25 years. What she does not say is that this compares with fewer than 50 cases of invasive cancer under age 25 years (compared to 440 aged 35–39 years). Given that screening has so little effect on incidence in this age group and the fact that the age-specific rate is not much greater anywhere in the world, we doubt that the number would have been appreciably larger in the absence of screening. Furthermore, the extremely small number of cancers prevented (if any) by screening under the age of 25 years must be balanced against the extremely high rates of cytological abnormality (15%) and unnecessary treatment, with a complication rate of cervical stenosis in up to 3% of those treated (Luesley *et al*, 1985).

There is no point in detecting precancer if this does not prevent invasive cancer. Cervical screening does detect very large numbers of CIN3 in young women, but the evidence is that it does little to prevent cancer. The reason must be that the vast majority of CIN3 in young women will not progress to cancer. Surely, it is better to start screening at the age of 25 years when many of the CIN lesions (including CIN3) will have regressed, but extremely few will have progressed to cancer; especially as there is no evidence that screening can prevent the rare but rapidly progressing lesions from becoming cancer.

Down-staging invasive cancers is indeed a useful effect of screening. However, an increase in microinvasive cancers that is not accompanied by a decrease in fully invasive cancer must be questioned. Our study shows that there is little or no reduction in fully invasive cervical cancer in women in their twenties associated with screening.

We remain confident of our findings and feel that the government is justified in changing their policy to only invite women for screening from age 25 years in England. No one would introduce a screening programme for a disease that effects fewer than 15 in 100 000 women (the cumulative rate of cervical cancer up to age 25 years, which has changed little since 1988), particularly when there is little evidence of its effectiveness and substantial evidence of harm.
